# Use of paracetamol in sows around farrowing: effect on health and condition of the sow, piglet mortality, piglet weight and piglet weight gain

**DOI:** 10.1186/s40813-021-00224-z

**Published:** 2021-08-07

**Authors:** Wikke Kuller, Steven Sietsma, Susan Hendriksen, Daniel Sperling

**Affiliations:** 1 University Farm Animal Practice, Reijerscopse Overgang 1, 3481 LZ Harmelen, The Netherlands; 2 CEVA Netherlands, Ceva Santé Animale B.V, Tiendweg 8c, 2671 SB Naaldwijk, The Netherlands; 3 CEVA Sante Animale, Havirna 1133, 67961 Letovice, Czech Republic

**Keywords:** Paracetamol, Sow, Piglet, Farrowing, Weight gain, Welfare, Immunocrit

## Abstract

**Background:**

Pain and fever in the periparturient period can lead to prolonged farrowing and can slow down the recovery of the sow, which will have an effect on the vitality and survival of the piglets. This study investigated the use of orally administered Paracetamol (Pracetam- CEVA) in sows in the periparturient period.

**Results:**

Mortality did not differ in piglets during the first week, or during total lactation (*P* > 0.10). No difference was found in weight or weight gain of the piglets during lactation. The coefficient of variation of piglet weight was smaller in the Paracetamol (Pm) group at day 7, day 14 and at weaning, but not at birth. So, the variation within litters was smaller in the Pm- treated sows, in comparison with the Control (C) litters. No difference in mean IgG concentration was found between treatments, but the coefficient of variation was too high (> 40) in 50% of the C litters and not in Pm litters. The Pm- treated sows lost less backfat than the C- sows. No effect was found on the body temperature of the sows, but fever was rare in both groups.

**Conclusion:**

Paracetamol results in less variation of body weight of piglets during lactation, seems to have a potential effect on the distribution of IgG within litters and has a positive effect on backfat loss. The effects of Paracetamol might be even more pronounced in farms with high piglet mortality (this farm only 8%) or with a high incidence of agalactia, fever after farrowing or piglet diarrhoea, which was not the case in this farm. Paracetamol is a promising product for increasing the welfare in lactating sows and optimising production in the farrowing stable.

## Background

The periparturient period is a critical period for both sows and piglets. It is generally accepted that farrowing is a painful process and thus can have adverse effects on the welfare of both the sow and piglets resulting in, potentially, a negative economic impact [1, 2]. Pain and inflammation accompanied by elevated body temperature in the periparturient period can lead to prolonged farrowing and can slow down the recovery of the sow after farrowing [3]. Any condition that will slow down post-farrowing recovery and discomfort of the sow will have an immediate effect on the vitality and survival of her piglets. Therefore, pain and illness associated with farrowing is a potential welfare concern. Inappropriate mothering style due to the pain and inflammation might influence the piglet’s behaviour, associated with reduction of colostrum/milk intake with consequent growth retardation and weakness, increasing the risk of crushing by the mother. For the periparturient period of the sow classical, non-steroidal anti-inflammatory drugs (NSAID’s) are frequently used (e.g. meloxicam, ketoprofen and flunixin) for their anti-inflammatory and antipyretic effect, as reviewed by Schoos [4]. Studies mitigating pain and inflammation using an NSAID showed variable effects on feed and water intake and constipation of the sow and growth and mortality in piglets [3, 5–9]. Side effects of NSAID’s, however, can be severe, if given over a longer period of time and many of them can only be administered parenterally and for a limited period of time [10, 11]. Several NSAID-based products are contraindicated during the peri-partum period due to the risk of adverse side effects.

Paracetamol (also known as Acetaminophen) is a medication of first choice after birth in human medicine due to the potent antipyretic and analgesic actions with limited side effects, especially in the case of its long term administration [12, 13]. In pigs, Paracetamol can be administered orally without any stress during the farrowing period. Oral administration in pigs is characterised by rapid absorption from the gastro-intestinal tract (GIT), with high systemic bioavailability up to 90%. Characteristically, it has a very low plasma protein binding (12.83 ± 3.11%) [14].

Due to the non-selective inhibition of COX1 and COX2, common NSAIDs have serious side effect on gastro-duodenal mucosa and the haematopoietic system. The most common result is erosion and ulceration of the mucosa, which is caused by non-selective inhibition of prostaglandin E2-mediated bicarbonate synthesis and mucus secretion with epithelialisation and local blood flow [15]. Paracetamol does not have such adverse side-effects on the gastric mucosa or haematological parameters, due to the selective effect on COX2 compared with other NSAIDs, which are used frequently in the field. The absence of gastrotoxicity of Paracetamol has been widely documented in animal and also human studies [12, 16]. The safety profile of Paracetamol is suitable for the initiation of treatment before farrowing in order to accomplish adequate plasma concentrations before, during and shortly after farrowing, minimising the risk of any negative effect.

Despite the clear potential benefit and safety profile of Paracetamol, studies evaluating the effect on the use of Paracetamol in the periparturient period in swine are missing. The aim of our study was to assess the effects of orally administrated Paracetamol in sows in the periparturient period. The effects were studied on general health and condition of the sow on the one hand and mortality, weight and weight gain of the piglets on the other hand. The possible effect of the medication on the immunity transfer to the piglets via colostrum was assessed, as well as the Paracetamol concentration in sow’s milk.

## Results

### Piglets

Most litters, 75%, were born on day 0 of the experiment. The other litters were born between − 1 and − 3 days before day 0. Piglets were weaned at 23.5 ± 0.9 days of age. Litter size at birth varied from 7 to 22 and averaged at 15.5 ± 3.0 piglets per litter.

**Fig. 1:**
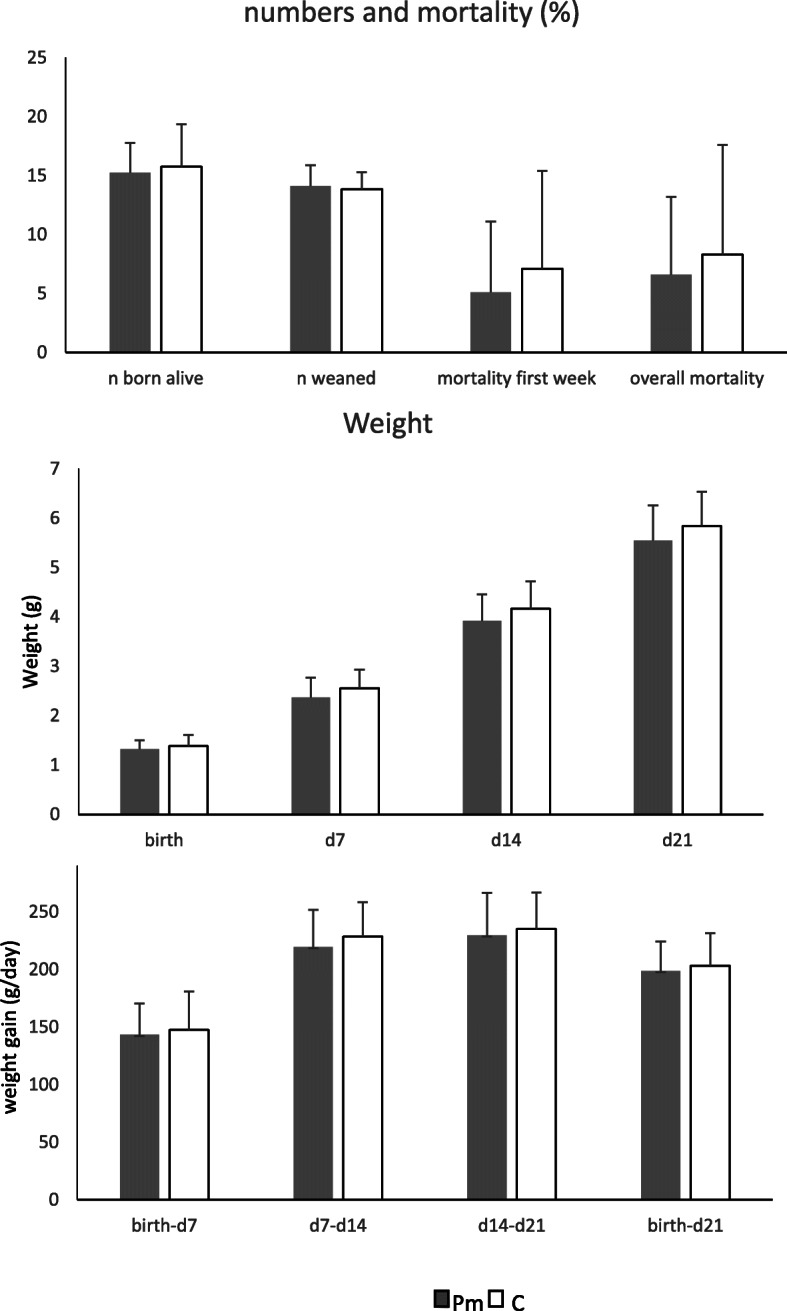
Numbers born and weaned, mortality, body weight and body weight gain (Means ± SD). Pm = Paracetamol, C = Control. The experiment started on day 0 and weaning took place at day 23. * *P* < 0.05

No significant difference was found between the number of piglets born alive and the number of piglets weaned (Fig. 1) between the Paracetamol group (Pm) and the Control group (C). Mortality did not differ significantly in the first week after birth (Pm: 5.1 ± 5.9% vs C:7.1 ± 9.2%; *P* = 0.40), or during total lactation (Pm: 3.5 ± 4.5 vs C:3.5 ± 3.1; *P* = 0.46). There was also no difference in the number of piglets cross fostered per litter (Pm: 6.6 ± 6.7% vs C: 8.3 ± 9.2%; *P* = 0.44), or in the percentage of piglets cross fostered per litter (Pm: 21.0 ± 23.0% vs C: 22.8 ± 19.7%; *P* = 0.66).

**Fig. 2:**
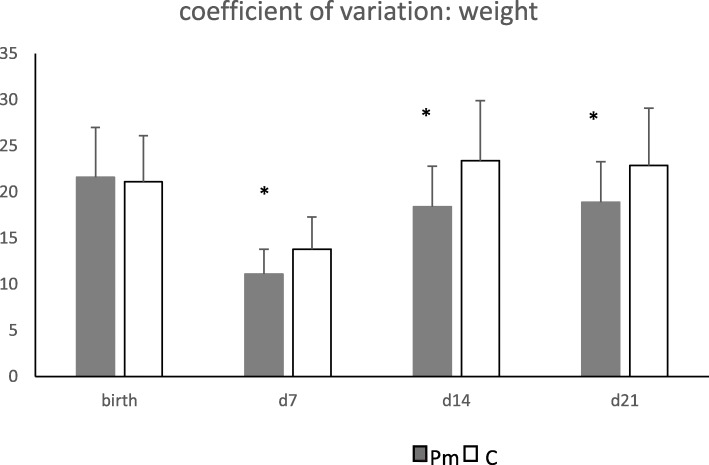
Coefficient of variation of body weight of the piglets. (Means ± SD). Pm = Paracetamol, C = Control. The experiment started on day 0 and weaning took place at day 23. * P < 0.05

No significant difference was found in body weight or body weight gain at any time during lactation (Fig. 1). However, a significant difference was found in the coefficient of variation (CV) or relative standard deviation of body weight of the piglets (Fig. 2). The CV did not differ at birth, but was smaller in the Pm group at day 7, day 14 and at weaning. So, the variation within litters was smaller and piglets were thus more uniform in the Pm group.

**Fig. 3:**
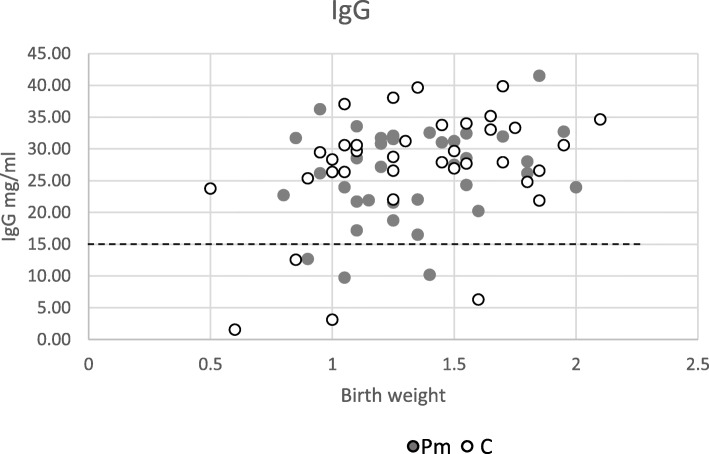
Birth weight vs IgG concentration (mg/ml) of newly born piglets. Pm = Paracetamol group; C = Control group. --- line indicates threshold line of IgG for survival

The mean IgG concentration in piglets was 26.1 ± 7.25 mg/ml in the Pm group and 27.4 ± 9.0 mg/ml in the C group (Table 1). The number of piglets with an IgG concentration less than 15 mg/ml (Fig. 3) did not differ between groups (Pm: *n* = 3 vs C: *n* = 4; *P* > 0.10). The coefficient of variation (Table 1) was higher than 40 in 50% of the C litters, which means that the variation was much higher. In contrast, the CV in the Pm group was more uniform with no CV value ≥40 observed.

**Table 1:** Coefficient of variation of IgG concentration. Pm = Paracetamol; C = Control

Treatment	Parity	Litter size birth	Mean IgG	Coefficient of variation IgG
Pm	10	18	22.7 ± 6.0	26.5
Pm	7	15	24.8 ± 8.4	34.0
Pm	6	13	33.9 ± 3.8	11.4
Pm	6	17	22.9 ± 6.6	28.9
Pm	4	12	26.4 ± 3.2	12.3
Pm	2	16	26.3 ± 9.5	36.1
C	9	13	30.3 ± 1.7	5.8
C	8	18	23.6 ± 5.7	24.3
C	7	14	21.8 ± 9.4	43.0
C	6	17	34.3 ± 4.8	13.9
C	3	12	29.4 ± 11.9	40.5
C	5	18	25.0 ± 12.2	48.6

Values are means ± SD.

### Sows

The number of sows that refused feed at least once during the 12 measurements did not differ between groups (Pm: 14 of 23 vs C: 13 of 21; *P* = 0.75). The number of sows that had a fever (T > 39.4 °C) did not differ between groups (Pm: 8 of 23 vs 11 of 21; *P* = 0.23). The percentage of faecal scores (12 per sow) with score 0 (no faeces) or score 1 (dry faeces) within each sow was high, but did not differ in between groups (Pm: 83.9 ± 3.4% vs. C: 87.0 ± 3.5%; *P* = 0.53). No significant difference was found in backfat measurement at farrowing (Table 2). However, the backfat thickness was significantly lower in the C group at weaning due to a higher backfat loss in group C sows during lactation.

**Table 2:** Back fat and backfat loss of the sows at farrowing and at weaning

	Paracetamol	Control
Number of sows	23	21
Backfat at farrowing (mm)	18.0 ± 3.6	18.3 ± 4.4
Backfat at weaning (mm)	13.2 ± 3.2^d^	12.4 ± 3.5^e^
Backfat change (mm)	- 4.8 ± 1.8^d^	- 5.9 ± 2.7^e^

^d,e^ Different superscripts in a row indicate differences between treatment (*P* < 0.05) Values are means ± SD

## Discussion

It has been recognised that animals feel and suffer pain in a similar manner to humans, consequently, the welfare benefit of its control is widely recognised. The NSAID class of drugs, together with Paracetamol, are commonly recognised as being very efficacious substances in swine medicine. Inflammation and trauma, which can be frequently associated with farrowing, have a negative impact on health, productivity and welfare in sows and consequently to their progeny. Parturition pain is associated with the release of opioids, which negatively influence oxytocin release and consequently milk let down [17, 18]. The indirect effect of anti-inflammatory treatment on the immune status of piglets was demonstrated, when higher concentrations of IgG during the first 48 h of life were observed in piglets from treated sows [3].

In the study, no significant effect was found on the mortality rate of piglets during lactation. This might be explained by the fact that mortality during the experiment was already very low, taking into consideration the average parameter on the selected farm was approximately 8%. Usually, the mortality rate during lactation in this farm, based on the assessment of the production records, was 10–11%, which is also low, compared to the Dutch standard of 13.5%. The low mortality found, might be an effect of the experiment itself, because there were extra co-workers in the peri-parturient period, compared with the usual on-farm standard. Other similar studies failed to demonstrate significant effects of therapy on pre-weaning mortality ([2] [3, 8]). In contrast, a positive effect on mortality reduction (*P* < 0.05) was observed in a study where sows were medicated with Paracetamol for four days but in this particular case, significant differences in rectal temperature, time lying down and daily feed intake were demonstrated as well, suggesting there may have been health issues in the control group [19]. Furthermore, for this pilot, a farm with consistent results in the farrowing stable was chosen, to reduce the chances that any additional circumstances (e.g. neonatal diarrhoea) would interfere with the experiment. This might be an explanation for the small differences between the Paracetamol and control groups.

No significant effects were found on bodyweight or weight gain of the piglets. So, even if the sows were feeling better in the peri-parturient period, this was not reflected in overall piglet’s weights or weight gain in this study. Previous studies demonstrated that treatment, which reduced inflammatory responses in the post-partum period, could increase milk yield [20]. No significant improvement of the bodyweight of piglets in the current study might be explained by the fact that there was no difficult farrowing observed, no long periods with elevated body temperature or any signs of infection of the mammary gland (the mastitis, metritis, agalactia (MMA) syndrome) recorded in both groups of sows and thus differences in milk yield were most probably not significant. It is well known that sows with fever and pain during lactation spent less time lying down, thus limiting time for piglets to suckle [21]. However, the coefficient of variation, a measure of relative variability of piglet body weights from day 7 onwards, was lower in litters of Paracetamol-treated sows. There was less variation in body weight in these litters (within litter variation) and thus litters were more equal in weight at weaning, providing possible future production benefits, as weaning weight is one of the important production predictors. This is in line with data from Mainau et al. [2] where piglets of low birth weight had higher average daily gain when sows were treated with meloxicam, suggesting that also in that study variation in body weight of piglets has decreased in sows treated with an NSAID. Most likely, milk is more equally distributed within these litters, which might explain the fact that there was less variation within litters in bodyweight. The positive effect might be supported by improved suckling ability of piglets and a better milk let down of the sow because of alleviation of pain. An assessment of the time, when sows were lying down, would help to explain the differences observed.

The possible improved suckling ability is supported by the fact that the variation in IgG in the blood, which reflects colostrum intake by the piglets, was much higher in the Control group. Although statistical analysis could not be performed because of sample size, it is remarkable that the coefficient of variation (CV) > 40 in 50% of the litters was observed in the control group. At a farm level, 20% of the best farms have a CV of IgG < 20 and the 20% worst performing farms have a CV of > 34 (personal communication In:Newsletter For Farmers, 2014). In line with our results, a study of Mainau et al. [3] also found a positive effect of meloxicam on IgG levels of piglets.

Despite the fact that sows in the control group did not experience any strong stress-inducing episodes, like prolonged farrowing, dystocia and consequent inflammation, differences in IgG CV may support the hypothesis of a post-partum inflammatory state in apparently healthy animals, which is described in dairy cows [22]. Studies and available information in sows are unfortunately missing.

Further assessment of general health and disease incidence in piglets would be interesting, as not only humoral immunity transfer happens via colostrum but as well acquired cellular immunity is in place. For the possible improvement of the immune transfer due to the treatment by NSAIDs, the timing of the treatment is especially important. Treatment before expected farrowing or at the onset of farrowing is considered crucial, as any delay could compromise any resulting possible benefits.

Neonatal survival and performance of piglets during the lactation period depends, on one hand, on the health and performance of the sow (e.g. speed of farrowing, lying behaviour, milk production) and on the other, the piglet’s characteristics (e.g. vigour, birth weight) [23]. Paracetamol might affect the health status of the sow, thereby alleviating the impact of the peri-parturient period. This might also result in more vigour of the piglets (easier farrowing process), with better colostrum and milk uptake as a result. However, since Paracetamol is transferred to the colostrum and milk, just like meloxicam [24], there might also exist a direct effect on the piglets during the first days of life. The data of this pilot study did not explain how the administration of Paracetamol affected the offspring.

Sows treated with Paracetamol lost less backfat than non-treated sows. This observation is aligned with previous reports, when for example, application of ketoprofen maintained backfat better during the lactation period compared with a non-medicated control group [8]. In the periparturient period, however, no differences were found in number of sows with feed refusal, nor in the percentage of sows with no or dry faeces, which could indicate differences in feed or water intake immediately after farrowing. Sows were not fed ad libitum in the peri-parturient period and the exact consumption of food was not measured and consequently the direct impact on improvement of feed intake cannot be assessed in the study. It is possible that these treated sows had a better and faster start of feed intake after day 2 of the experiment, when the farmer started increasing the portions to 7.5 kg during a period of 10 days. A similar observation was recorded, in a study assessing the effect of peri-parturition use of Paracetamol premix, when a significant increase (*P* < 0.05) of daily feed intake was observed during the first 3 days after farrowing in the medicated group [19]. Another study confirmed that the appetite of sows was significantly less disturbed in the batch treated by Paracetamol than in the control (Chi 2 test, *p* = 0.033) [25]. Farrowed sows expressed reduced feeding for longer period when pain was not treated by ketoprofen, as well hyperalgesia causing behavioural changes lasting up two days post trauma was described in a rodent model [8, 26]. This could explain the fact that animals treated with Paracetamol had a less negative energy balance during lactation. A potential, positive effect on subsequent onset of oestrus and fertility was not studied in this experiment, or the welfare impact on the prevalence and severity of shoulder sores [18].

In this study, treating sows with Paracetamol in the peri-parturient period resulted in less variation within litters in the bodyweight of piglets. However, the effects of Paracetamol are most probably more pronounced in farms with higher mortality and thus less optimal farrowing and lactation management. Effects might also be more pronounced, if only sows at risk of lactation problems were compared to healthy animals. More research is also needed on how the beneficial effects on piglets, after administering Paracetamol to sows, is accomplished.

## Conclusions

Paracetamol has a positive effect on backfat loss during lactation, without any negative effects on mortality or weight gain of the piglets. Moreover, this pilot showed a positive effect on variation of IgG concentrations and on variation of piglet weight at weaning. Loss of backfat and thus negative energy balance of the sow is one of the health parameters of sows during lactation. Paracetamol is considered a promising product for improving the health and welfare of lactating sows and their piglets in the periparturient period, but more research is needed, especially on farms with more severe periparturient problems.

## Methods

### Animals and housing

Forty-four sows (Topigs TN70) from one farm in one farrowing batch were included between April and May 2019. All sows farrowed in the same week, divided into two farrowing houses. The sow’s parity ranged from 1 to 10 and was 4.3 ± 2.6 on average. During lactation, the sows were housed individually in pens in farrowing crates (Nooyen Balance Floor; 0.56 × 2.17 m) in accordance with Dutch legislation. The farrowing pen consisted of 1.13 m^2^ solid floor and 3.35 m^2^ slatted floor. At one side of the pen there was a piglet nest (0,45 × 1,81 m) with an infrared lamp and floor heating. Sows were fed a commercial lactational feed starting at a level of 3 kg at the start of lactation to 7.5 kg within 10 days after farrowing, divided into two meals a day. Water was available ad libitum. During gestation, sows were vaccinated to prevent diarrhoea in suckling piglets (Porcilis Coliclos, MSD Animal Health) and all sows were regularly vaccinated against *Erysopelothrix rhusiopathiae* (Porcilis Ery, MSD Animal Health).

Most litters (33 of 44) were born on Tuesday and therefore this day was designated as day 0 of the experiment. Cross fostering was performed by the farmer based on litter size, piglet weight and parity of the sow and only within their respective treatment group. At day of birth, piglets were ear tagged according to Dutch legislation and for individual identification. Within 3 days after birth, piglets received an injection with 1 ml iron (Dextran 20%, MS Schippers). At day 21 of the experiment all piglets were vaccinated against Circovirus (Ingelvac CircoFLEX, Boehringer Ingelheim). Piglets were creep fed from day 7 onwards. Drinking nipples were used to give ad libitum water to the piglets.

### Treatments

Sows were stratified based on parity and then randomly allocated to treatment or control. Animals allocated to the control group were not treated, because no placebo was available. A total of 23 sows were allocated to the Paracetamol group and 21 sows to the control group. In the treatment group, sows were given total 20 ml of Paracetamol (400 mg/ml) over their feed, divided over two meals (6.15 a.m. and 17.00 p.m.) each day. Treatment started for all sows at experimental day − 3 and was continued until two days after farrowing (Table 3). In the Paracetamol group 2 sows farrowed earlier than experimental day 0 (at 1 and 2 days before day 0 respectively), so all sows had at least 2 treatments of Paracetamol before the start of farrowing. The study was double-blinded with the allocation to treatment group and the treatment performed by an independent technician.

**Table 3:** Time line of the experiment

Experimental day		Treatment
−4		Sow: BF
− 3	Paracetamol	Sow: T, FS and FI New born piglets: BW and ET
−2	Paracetamol	Sow: T, FS and FI New born piglets: BW and ET
−1	Paracetamol	Sow: T, FS and FI New born piglets: BW and ET
0	Paracetamol	Sow: T, FS and FI New born piglets: BW and ET
1	Paracetamol	Sow: T, FS and FI Piglets: BS
2	Paracetamol	Sow: T, FS and FI New born piglets: BW and ET
7		Piglets: BW
14		Piglets: BW
21		Sow: BFPiglets: BW

BF = Backfat; T = Rectal Temperature; FS = Faecal Score; FI = Feed Intake; BW = Body weight; ET = Ear Tag; BS = Blood Sampling.

D0 = most litters were born on Tuesday, so this day was designated as day 0 of the experiment.

### Measurements

*Piglets*- Piglets were weighed individually at birth, day 7, day 14 and day 21. Average daily gain was calculated. At day 1 after birth, blood samples were taken of 6 piglets (two lightest, two middle, two heaviest piglets) of 6 litters in each treatment (72 piglets in total, 36 in the treatment group and 36 in the control group) in order to assess colostrum intake (Immunocrit method). For blood sampling, litters were selected, based on being born on day 0 and from sows with parity 2 or higher. General health parameters were checked daily by the farmer. Use of medication was monitored.

*Sows*- Backfat thickness (50 mm from the midline over the last rib) was measured at day − 4 of the experiment and at day 21. Rectal temperature, feed intake (0 = non-eater, 1 = eating less than schedule; 2 = normal eater; 3 = eating above schedule) and faecal scores (0 = no faeces; 1 = dry faeces; 2 = normal faeces; 3 = pasty faeces) of the sows were assessed twice daily from day − 3 to day 2 of the experiment. Feed intake and faecal score was always assessed by the same technician. General health parameters were checked daily by the farmer. Use of medication was monitored.

### Immunocrit

All 72 samples were tested by the Immunocrit test [27] in the laboratory of the University Farm Animal Practice. To perform the Immunocrit test, 50 μl serum is mixed with 50 μl 40% (NH4)2SO4 (ammonium sulphate). The mixture was inserted into a haematocrit capillary tube and centrifuged for 10 min at > 13.000 G. The ratio of the mm precipitate to mm solution in the tube is measured, and used to calculate an Ig concentration, based on earlier defined and validated standards of the laboratory.

### Statistical analysis

All data were tested for normality using the Univariate procedure of SAS (SAS Inst. Inc., Cary, NC). Data were analysed using the GLM procedure of SAS. Body weight, weight gain and mortality were analysed as litter characteristics (*n* = 44). Data are presented as means ± SD. Differences are considered to be significant if *P* < 0.05. Relevant two-way interactions were not significant.

The following model was used to analyse data in the PROC GLM procedure of SAS:

$$ \mathrm{Y}=\upmu ={\mathrm{T}}_{\mathrm{I}}+{\mathrm{LS}}_{\mathrm{j}}+{\mathrm{P}}_{\mathrm{k}}+{\mathrm{e}}_{\mathrm{ijk}} $$

Where Y = parameter studied (e.g. weight gain), T_i_ = treatment, LS_j_ = litter size, P_k_ = parity of the sow and e_ijk_ = error term of the model.

In the model for weight (at day 7, day 14 and day 21, not at birth), weight gain and percentage of animals cross fostered also birth weight was included as a covariate. In the model for backfat of the sow at weaning and back fat change during lactation also back fat at farrowing was included as a covariate. Differences in the number of cross fostered piglets and the number of sows with fever were tested using the χ^2^- test in the FREQ procedure in SAS.

## Data Availability

The data that support the findings of this study are available from the authors upon request.

## Abbreviations

BF: Backfat
BS: Blood Sampling
BW: Body weight
C: Control
CV: Coefficient of Variation
D0: Day 0 of the experiment
ET: Ear Tag
FI: Feed Intake
FS: Faecal Score
Pm: Paracetamol
T: Rectal Temperature
